# Man-Machine Interface System for Neuromuscular Training and Evaluation Based on EMG and MMG Signals

**DOI:** 10.3390/s101211100

**Published:** 2010-12-07

**Authors:** Ramon de la Rosa, Alonso Alonso, Albano Carrera, Ramon Durán, Patricia Fernández

**Affiliations:** 1 Laboratory of Electronics and Bioengineering, ETSI de Telecomunicacion, Universidad de Valladolid, Campus Miguel Delibes, Paseo Belén, 15. 47011 Valladolid, Spain; E-Mails: alonso3@tel.uva.es (A.A.); albano.carrera@uva.es (A.C.); 2 Optical Communications Group, ETSI de Telecomunicacion, Universidad de Valladolid, Campus Miguel Delibes, Paseo Belen, 15. 47011 Valladolid, Spain; E-Mails: rduran@tel.uva.es (R.D.); patfer@tel.uva.es (P.F.)

**Keywords:** biological control systems, training, pattern classification, electromyography, mechanomyography, real-time systems, sensors

## Abstract

This paper presents the UVa-NTS (University of Valladolid Neuromuscular Training System), a multifunction and portable Neuromuscular Training System. The UVa-NTS is designed to analyze the voluntary control of severe neuromotor handicapped patients, their interactive response, and their adaptation to neuromuscular interface systems, such as neural prostheses or domotic applications. Thus, it is an excellent tool to evaluate the residual muscle capabilities in the handicapped. The UVa-NTS is composed of a custom signal conditioning front-end and a computer. The front-end electronics is described thoroughly as well as the overall features of the custom software implementation. The software system is composed of a set of graphical training tools and a processing core. The UVa-NTS works with two classes of neuromuscular signals: the classic myoelectric signals (MES) and, as a novelty, the myomechanic signals (MMS). In order to evaluate the performance of the processing core, a complete analysis has been done to classify its efficiency and to check that it fulfils with the real-time constraints. Tests were performed both with healthy and selected impaired subjects. The adaptation was achieved rapidly, applying a predefined protocol for the UVa-NTS set of training tools. Fine voluntary control was demonstrated to be reached with the myoelectric signals. And the UVa-NTS demonstrated to provide a satisfactory voluntary control when applying the myomechanic signals.

## 1. Introduction

There is a relevant background related to neuromuscular signals applied to Rehabilitation Technologies. Myoelectric signals (MES) have been proposed to command devices, such as robotic prostheses, or act as generic man-machine interfaces in many papers [1–5]. But prosthesis adaptation is a major drawback for the limb impaired [6]. Amputees usually believed that a neuromuscular prosthesis could easily and completely replace the functions of the biological limb. However, direct interaction with a robotic prosthesis can produce unsuccessful results, cases involving children being the most sensitive ones [6]. As a consequence, the acquisition of myoelectric prostheses has decreased drastically. Prior to its utilization by a patient, it is necessary to evaluate the user’s ability to accommodate the prosthesis. Hence, a training and adaptation process is required.

MES processing techniques have been widely analyzed and they continue under research. The classical analog processing systems have been very reliable in rehabilitation robotics [7]. However, since some years ago, improvements in signal processors rely on digital processing [8–10]. But the embedding of robust digital processors in Rehabilitation Technologies is a recent field up till now.

Several papers have been published analyzing the myomechanic signals (MMS), usually denoted as the mechanomyogram (MMG) of a muscle. These works have proven a close relationship between muscle voluntary activity and MMS [11–13]. From an electromagnetic point of view, the sensitivity of MES to electromagnetic interference (EMI) has been established [7]. In addition, it has also been stated that other biopotentials cause the contamination of the electromyogram (EMG) signal [14]. Due to its mechanical nature and the required signal conditioning, MMS offers a substantial robustness against EMI and biopotential contamination in comparison to MES. However, as far as we know, MMS has been hardly used as a man-machine interface to control a device. Hence, in this paper, we introduce an operative MMS sensor, designed and implemented specifically for this task.

MES and MMS studies are frequently presented as off-line analyses, developed after the signal recording. The expected next stage would be the analysis in a real-time system, as Hogan did in his classic work with his analog processor[15,16]. But there is a significant cost in time and resources to prepare a real-time hardware system, e.g., a robotic arm, for analog and digital processing.

To address the aforementioned issues, this paper introduces the University of Valladolid Neuromuscular Training System (UVa-NTS). The UVa-NTS is a real-time hardware and software multifunction platform; it is intended for research, simulation, virtual training and generic neuromuscular man-machine interfacing.

The important concept in this platform is to merge the real-time paradigm with advanced signal processing in neuromuscular signals. Nowadays, most of the new digital signal-processing techniques are still being evaluated off-line [9,17,18]. The main reason could be the powerful tools available for off-line analysis and processing, such as the widespread Matlab software. However the embedding into a real-time system is not straightforward. Then, the performance of the digital processing techniques in a real-time environment is still not fully demonstrated. Hence, the classical and efficient analog signal processing is still valid for practical purposes, as the interactivity with the user is a key factor.

The standard procedure to analyze neuromuscular signals is to work with a bioelectric amplifier and record the signals. A certain protocol is defined to generate the signals from the patient. And once a set of signals is achieved, they are off-line processed with a signal processing tool, e.g., Matlab, to analyze and display the results.

Multi-purpose proposals of interactive platforms can be found [19], but the gap between the advanced off-line processing and the real-time application is often left out. A vague justification related to the required time constraints for interactive work is offered to support the real time capabilities of the signal processing technique. The full integration of all the involved facts, *i.e.*, hardware, software and processing is not often implemented to demonstrate the real-time performance. Hence, the interactive visual feedback from the user is not fully achieved in the proposed systems.

This platform aims at reducing this gap, as the hardware and software solutions are fully integrated in order to optimize real-time behavior. Then, all the involved facts can be tuned-up jointly in order to perform interactively with the user. The platform was presented in the Virtual Rehabilitation meeting in Vancouver in 2008 [20], where several users successfully tested the Myo-Pong game performance in that meeting.

The purpose of this paper is to explain the techniques and the underlying architecture that allows the interactive performance. It outlines the UVa-NTS hardware and software structure, as well as some of the tests carried out to evaluate its performance. A fully functional UVa-NTS prototype was implemented and results were obtained both statically and dynamically. These results were achieved with MES and MMS, and also both with healthy and impaired subjects.

## 2. Description of the System

**Figure 1.**
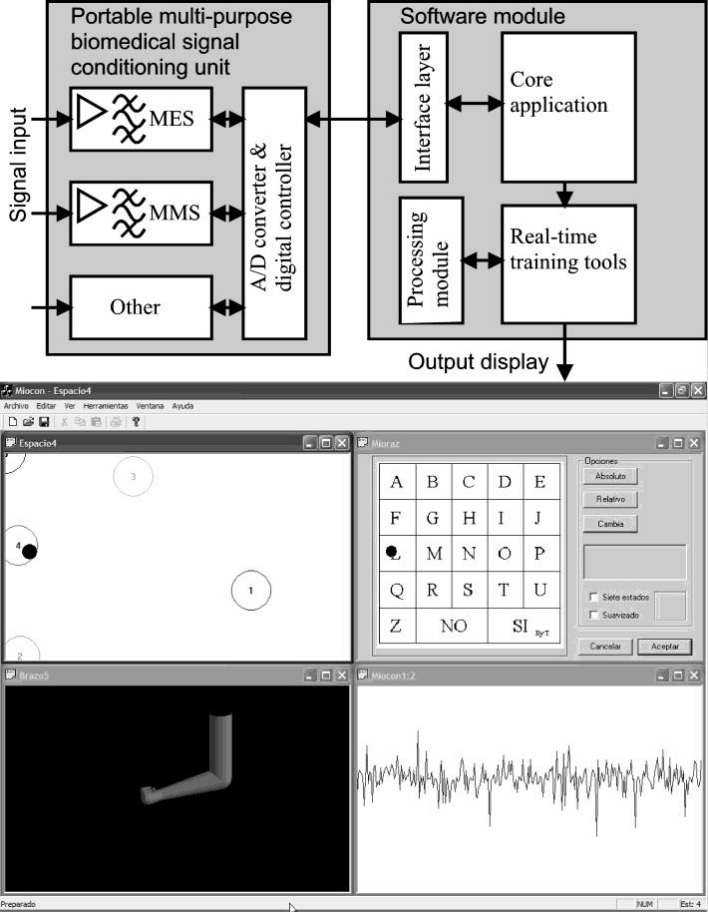
Block diagram of the UVa-NTS which is composed of a hardware signal-conditioning unit, a software module running on a PC and a graphical user interface (GUI). Tools displayed on the GUI: State navigator (top left), virtual keyboard (top right), virtual arm (bottom left), signal viewer (bottom right).

The UVa-NTS can be divided into two different parts: the hardware signal-conditioning unit and the control and processing software which runs on a personal computer (PC). As shown in Figure 1, the power and flexibility of the system software is based on merging four key concepts: (i) isolation from the hardware level by means of an interface layer, (ii) a processing module, (iii) a real-time software core, and (iv) a set of evaluation tools.

### 2.1. Hardware Module

**Figure 2.**
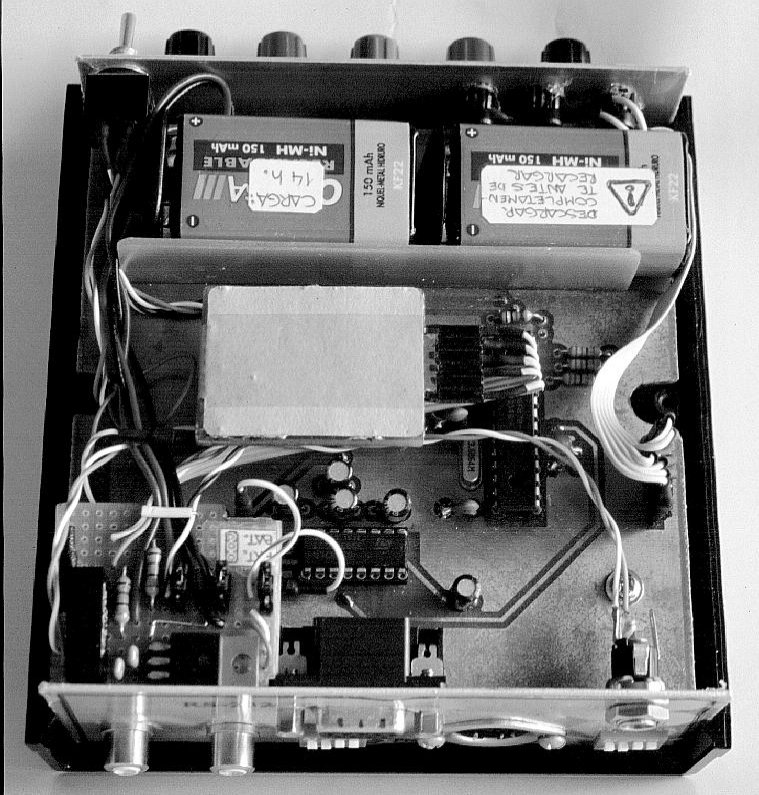
Assembled unit for signal-conditioning and communication with the computer.

Figure 2 shows a picture of the custom and portable signal-conditioning unit developed for this system. The unit is composed of bioelectric and biomechanical amplifiers. The former are implemented with instrumentation amplifiers and compact 8th-order switched capacitor filters, while the latter are composed of pump-charge amplifiers for the MMS piezoelectric vibration sensors. Then, a microcontroller performs the analog-digital conversion (ADC).

**Figure 3.**
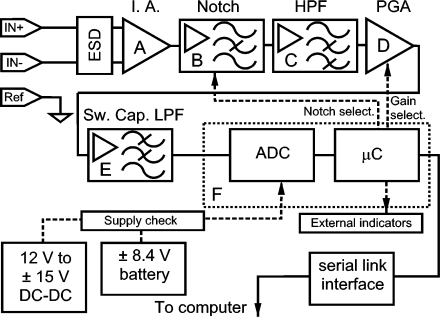
Signal conditioning electronics: front stage of the system. ESD: Electrostatic Discharge Protection. I.A.: Instrumentation Amplifier. HPF: High-Pass Filter. PGA: Programmable Gain Amplifier. Sw. Cap. LPF: Switched-Capacitor Low-Pass Filter. ADC: Analog to Digital Converter. μC: microcontroller.

The block diagram of the front stage is shown in Figure 3. This front stage is combined with digital signal processing in the computer. It will afford a 20 Hz to 400 Hz band-pass with a 60 dB/octave roll-off at 20 Hz and 22 dB/octave at 400 Hz, which constitutes a better approach than De Luca’s recommendations of 12 dB/octave [21]. Common Mode Rejection Ratio (CMRR) is better than 118 dB at 50 or 60 Hz and its gain can be selected between 74 dB and 89 dB.

Bioelectric signal acquisition requires voltage gains between 1,000 and 10,000 [22] or, in dB, between 60 dB and 100 dB. Highest gains are oriented to electroencephalogram, as the surface MES ranges are comprised between 5 μVrms and 300 μVrms [7]. It is important to connect gain with the input dynamic range of the analog to digital converter (ADC), to take advantage of the digital resolution of the ADC. Two gains have been considered from tests in the front stage in order to detect efforts: 74 dB and 89 dB, related to an input dynamic range of ±2.5 V with uniform quantization. Gain has been divided into several stages in order to prevent saturation due to excessive gain in a single block, and also to compensate for voltage offsets due to direct coupling of stages, avoiding offset trimming.

#### 2.1.1. Instrumentation Amplifier

Surface MES is acquired by means of a single differential electrode with two contacts and a reference. The preamplifier is implemented with an integrated instrumentation amplifier INA114 [23] with a fair input bias current I_bias_ of 2 nA. This is configured with a gain of 34 dB that yields a CMMR better than 115 dB at 50 Hz or 60 Hz.

**Figure 4.**
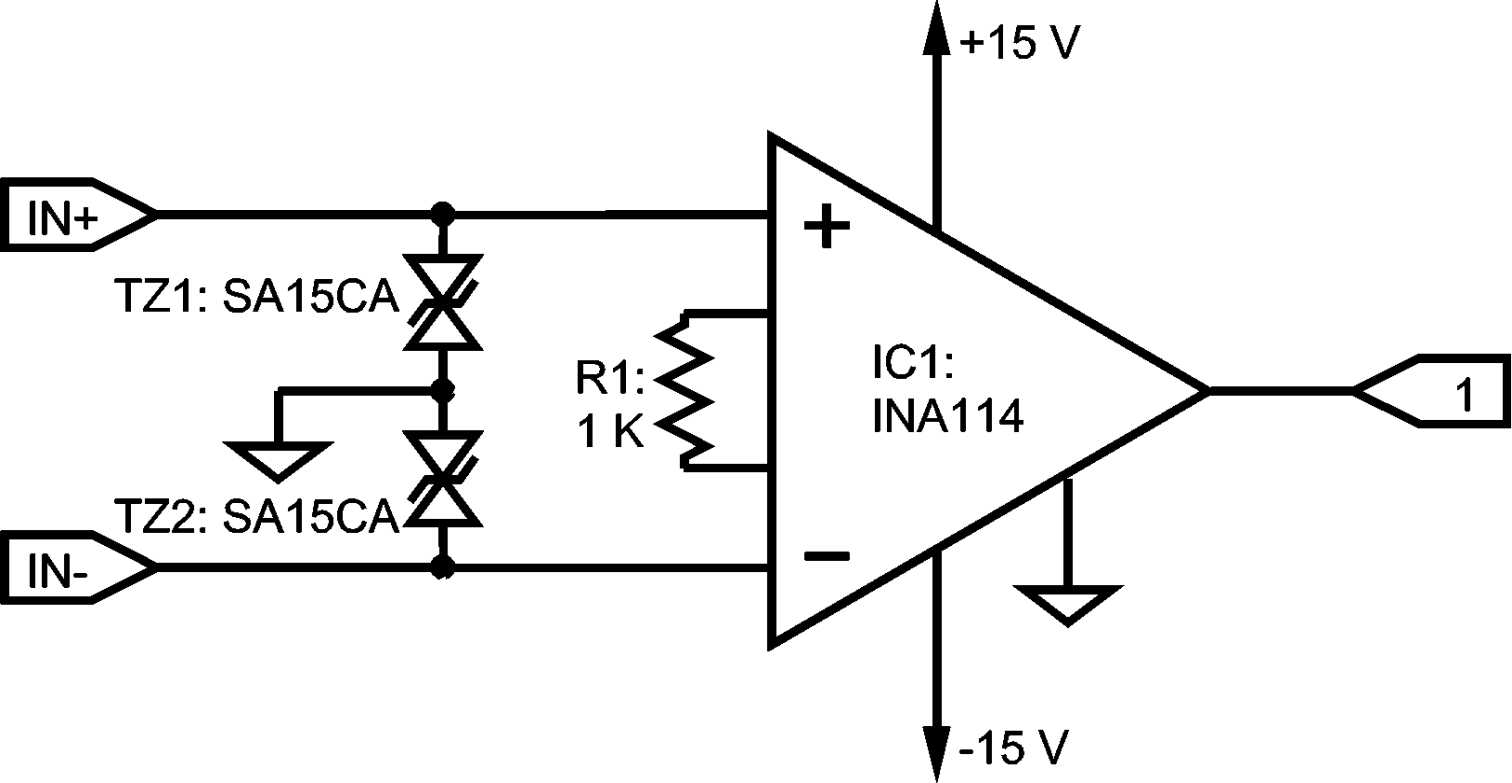
INA 114 instrumentation amplifier with ESD protection; gain = 34 dB, to be assembled near the electrode for differential electrode configuration. Label #1 at the output connects to the notch filter input.

Figure 4 shows the schematic of the small circuit which can be located near the electrode to reduce noise and interference, such a location is significant with dry electrodes and with a poorer interface skin-electrode [7,21]. The selected IA incorporates an over-voltage input protection of ±40 V. Since those inputs are often handled with the risk of degradation due to electrostatic discharges (ESD) [23–25], SA15CA bi-directional transient suppressors with 15 V clamping voltage [26] have been included.

The goal is to achieve an overall gain of 74 dB or 89 dB. When IA is configured to 60 dB it yields a remarkable 120 dB CMRR, but the output is saturated quite often for a supply of ±15 V. A satisfactory behavior was achieved with a 40 dB gain working with Ag-AgCl electrodes in a previous prototype: it was utilized for several years and the saturation differential voltage was near 150 mV for ±15 V supply. Saturation levels depend heavily on the power supply voltage, input impedance imbalance and I_bias_. Saturation phenomena increased when the front-end was operated with batteries, as supply was reduced to ±8.1 V. Hence, the gain was set to 34 dB to improve reliability with a saturation voltage near 300 mV for ±15 V supply. Under normal operational conditions, the device is supplied from mains with an external transformer giving a regulated 12 V DC, and a TMA1215D DC-DC converter from 12 V to ± 15 V that adds 1 kV of galvanic isolation [27].

#### 2.1.2. Notch Filter

**Figure 5.**
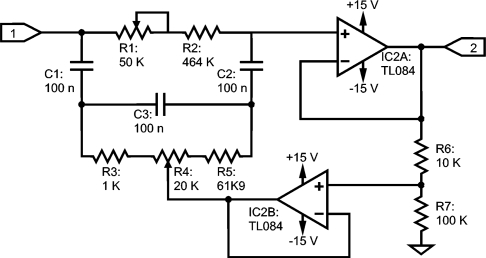
Trimmable bootstrapped twin-T as a notch filter for 50 Hz or 60 Hz; label #1 denotes input from the IA stage and label #2, output to the high-pass filter.

The ElectroMagnetic Interference (EMI) from the power line can be up to 100 dB stronger than the MES[7] and most of it is removed by the high CMRR of the IA. However, a certain differential voltage that is amplified still appears. To remove the power line EMI, digital processing is available after the ADC, but the whole gain is applied when the signal reaches the input of the ADC. Hence, a notch filter has been inserted prior to the high-pass stage and before applying the remaining 40 dB or 55 dB to both avoid dynamic range reduction and to achieve the total gain. The filter is a tunable twin-T stage from 25 Hz to 100 Hz [24], as shown in Figure 5, with a set up that works with multiturn trimmer pots. As it can substantially modify the frequency response, its activation is optional and it can be enabled by means of an MAX333 electronic switch.

#### 2.1.3. High-Pass Filter

**Figure 6.**
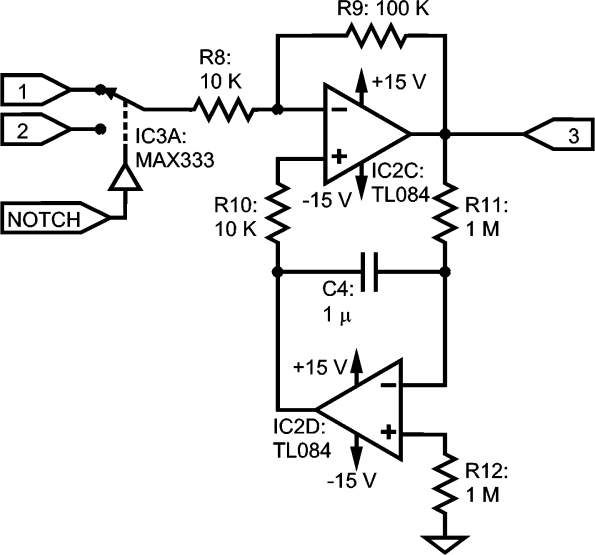
High-pass filter with a gain of 20 dB in the passband. Label #1: from IA; #2: from notch filter; #3: HPF output.

As a high-pass filter (HPF), a circuit variant from other designs already described in [22,23,28,29] is proposed. Our circuit is an independent module, as shown in Figure 6, separated from the IA structure. It is based on an inverting stage with the feedback of an integrator, yielding a 20 dB gain in the pass-band. This design avoids introducing new electronic components to the IA, so it can be located near the electrode and, in addition, it provides a new stage for gain distribution. DC-offsets from previous sections (IA, notch filter) are eliminated at the output by the high-pass behavior of the stage. Thus, the next section will have less gain requirements and this will give as a result a lower amplification of the residual output DC-offset of the HPF.

Resistors R11 and R12 must be equal to avoid excessive output DC-offset due to input bias currents of IC2D, and low I_bias_ operational amplifiers should be used, such as the four-to-a-package TL084, with I_bias_ = 30 Pa [30]. In previous tests with an LM324, an I_bias_ equal to 45 nA [31] was used as a starting point for this application [32], but it gave excessive offset when grounding the non-inverting pin.

#### 2.1.4. Programmable Gain Stage

**Figure 7.**
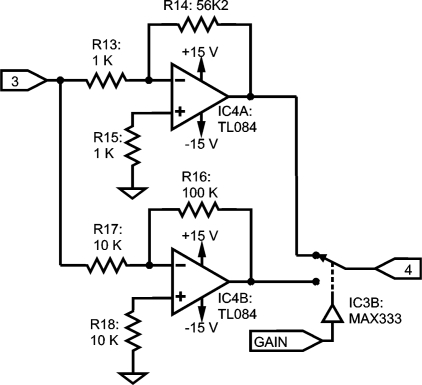
Selectable gain stage: 20 dB or 35 dB; it uses two of the four on-chip operational amplifiers.

Two stages in a standard inverting configuration have been set, as shown in Figure 7. Compensation resistors are used to reduce errors from input bias current [24], and gain selection is commanded by means of a MAX333 electronic switch.

As the previous stage (Figure 6) has 20 dB with self-compensated DC offset, moderate gains are used here, *i.e.*, 20 dB and 35 dB, in order to avoid excessive amplification of undesired DC-offset from the previous stage.

#### 2.1.5. Low-Pass Continuous-Time and Switched Capacitor Filters

To minimize the signal distortion, special care must be taken with the band-pass in a sampled system. The Nyquist Sampling Theorem states that a band-limited signal is uniquely determined by its samples if the sampling frequency exceeds twice the upper frequency limit of the band. Otherwise, frequency conversion and spectral aliasing are produced, originating distortion in the reconstructed signal [33–35]: this can interfere with features such as the frequency displacement associated to muscle fatigue [21,36,37]. Attention was paid to the frequency response, both in magnitude and phase. Hence, bandwidth limitation has been implemented with a Bessel-Thomson configuration. In comparison with other configurations, the Bessel-Thomson filter maximizes phase linearity, at the expense of a smoother magnitude response [38].

**Figure 8.**
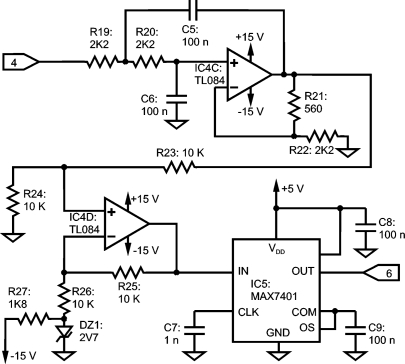
Anti-aliasing low-pass Bessel filters: continuous and switched capacitor filter.

A Bessel switched capacitor filter (SCF) is used in the design, but an analog continuous-time filter previous to the SCF is mandatory: internally, the SCF consists of a sampled system, so the Nyquist Sampling Theorem must be taken into account. The continuous-time filter implemented is a voltage-controlled voltage-source filter [24]. A second order Bessel configuration is implemented, consisting of IC4C, R19, R20, R21, R22, C5 and C6 as shown in Figure 8. The SCF is made up with an 8th-order Bessel filter MAX7401, completed with C7, C8 and C9. The remaining components of Figure 8 describe a 2.7 V level shifter to accommodate the symmetrical signal range to the 0 V – 5 V ADC input dynamic range.

#### 2.1.6. Analog to Digital Interface

A PIC16F873 microcontroller with an embedded 10 bit ADC is the core of the digital interface with the computer. It is endorsed with a 3.6864 MHz crystal for minimum error in the baud rate generator [39], achieving a throughput of 57,600 baud in the serial transmission. The ADC has its own timing, running independently of the crystal. It achieves a 1 kHz sampling rate per each of the two MES channels, being able to sample a single channel at 2 K-samples/s.

Two additional inputs of the ADC are used to sample the level of the symmetrical power supply by means of an arrangement of resistors. A MAX232 is employed as a chip interface to a serial RS-232 link to the computer, and a RS-232 to USB adapter allows switching between the two standards.

In the prototype, the SCF and the digital stages are powered by means of a standard 7805 voltage regulator, so it can work with batteries or from mains. However, to complete galvanic isolation, a DC-DC converter TMA1205S must be used. Isolation in the computer link is achieved by means of a standard RS-232 optical isolator.

### 2.2. Software Subsystem

The UVa-NTS was designed to be adapted to different signal sources, as necessities appear. To support this philosophy, a software interface layer between the signal-conditioning unit and the core application was created. It permits the connection of new devices to the PC, and only minor changes are required in the global software application. Most of the changes will be focused on the aforementioned interface layer, which is developed in C++ and compiled to a shared library.

The UVa-NTS software core and the evaluation tools were developed in C++. A formally non-strict (interactive) real-time application [40] was designed. Matlab was chosen as the engineering language to implement the processing module. It was selected due to its processing capabilities and its application in related works [9,17,41]. The Matlab code was compiled to C to accomplish the real-time response of the UVa-NTS. Subsequently, the processing module was compiled to a shared library. As a result, fast and complex processing algorithms can be created and distributed with no need of Matlab in the target computer.

The set of the UVa-NTS evaluation tools are displayed on a graphical user interface (GUI), as shown in Figure 1. Nowadays, these test and training tools are: a signal viewer, a state navigator, a virtual arm, a virtual keyboard and a version of the classical *Pong* videogame, called Myo-Pong. New tools can be implemented, with the support of the aforementioned C/C++ modular framework.

#### 2.2.1. Signal Viewer

This tool displays the amplitude/time representation of the involved signals, e.g., the EMG for MES or the MMG for MMS. A calibration procedure is required to adapt the system to the user. The signal viewer is the starting point in this procedure: gain and electrode contacts are tested before starting with the rest of the tools and signal amplitude dynamic range is visually checked.

#### 2.2.2. State Navigator

The state navigator is a two-signal source tool. It works with signals from two sources, commonly muscles. We have selected MES and MMS from two muscles in the present experiments. Horizontal axis represents the activity of one selected muscle, and vertical axis, the other selected one. The calibration procedure requires the two-channel calibration. The complexity of the calibration procedure depends on the processing algorithm, as two processors were developed for this system: an envelope detection processor and a pattern recognition processor.

The implemented envelope processor requires two steps. The first calibration step is in a rest situation in order to detect background noise. The second step requires an important effort to set up the signal dynamic range. Maximum voluntary contraction (MVC) is not needed, but it should be sustainable and comfortable.

The custom pattern recognition processor defines five or seven reference states. The number depends on the UVa-NTS operator ability, as state separability is reduced with the number, and UVa-NTS operation is more challenging. These states are related to different combinations of contraction levels from two muscles. The recognition processor is based on the conditional probability density function estimation and classification[42]. The density function estimation is obtained from reference patterns during the calibration procedure. Feature extraction is performed during real time operation. Thus, the calibration complexity is increased in comparison with the envelope detector.

Once the state navigator is calibrated, the operator trains the muscle voluntary control by displacing the black pointer among the different states. These states are numbered and encircled as displayed in Figure 1. The recognition processor makes real-time decisions with the feature vectors, and chooses the most suitable state, e.g., the nearest one in distance. To improve the user adaptation, the position of each state can be displaced to a more comfortable one during the operation of the system. The state navigator is considered as the previous step before managing the virtual arm tool.

#### 2.2.3. Virtual Arm

It is an anthropomorphic representation of a human arm. It is modeled with the biomechanical human joint restrictions. It is capable to open/close the hand, pronate/supinate it and extent/flex the arm. It serves as a prosthesis trainer for amputees or as an augmentative trainer for the disabled with reduced mobility. It is closely connected with the state navigator tool, because the encircled states, shown in Figure 1, act as reference positions to control the virtual arm. Then, each circle has a predefined action assigned, so when the pointer is situated on circle #4, the hand closes; when it is displaced to #3, the hand opens, and so on to extent/flex the arm or to pronate/supinate. The action set depends on the selected number of states. The action speed can be adjusted, depending on the dexterity of the user: for an experienced user, movements can be more responsive. The virtual arm was implemented with the OpenGL library achieving a very efficient rendering for real-time behavior, with a moderate programming effort.

#### 2.2.4. Virtual Keyboard

This tool works with two inputs from two muscles. Efforts from one muscle displace the pointer horizontally. The other muscle is employed for vertical movements. The selection is made by means of a quick double contraction. Selected letters are displayed on a box to compose words and sentences.

#### 2.2.5. Myo-Pong Game

**Figure 9.**
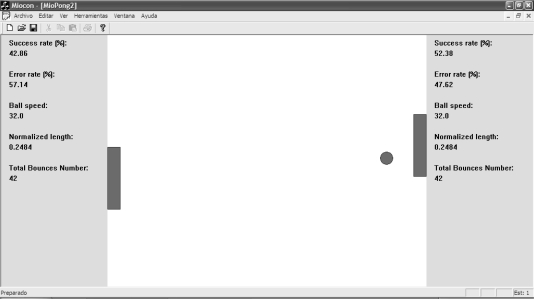
Appearance of the MyoPong game tool.

The last tool is the Myo-Pong (Figure 9), a table-tennis game that resembles a popular game in the 70 s, but applied to myoelectric control. It can be played by one player or by two players. The MES/MMS from two muscles are used to control the game. The lack of activity in the muscle places the bar (paddle) at the top of the screen, while the maximum voluntary contraction, calibrated for each muscle, causes the bar to slide to the bottom of the window. Also, there is a training mode and a game mode. In the former, there is no penalty when a hit is missed: the ball bounces on the side of the window and the game goes on. In the latter, the game goes on in the classic way, so the missed hits are penalized.

The tool allows changes in the size of the paddles and the speed of the ball and it registers the success and the error rate as evaluation parameters. Thus, a set of parameters to be measured was defined: the *Success Rate*, the *Admissible Speed*, the *Precision Control* and the *Fatigue Time* [43].

#### 2.2.6. The Processing Core

The present system is capable of performing the signal processing by means of envelope detection and also by pattern recognition. The former gives an analog feeling to the tools while the latter permits discrete state detection and allows multiple actions with a reduced number of signals. The combination of both techniques is tested in the set of implemented training tools.

The envelope detection processor has been widely analyzed in literature [15,16,44,45]. Thus, we will focus on the implemented pattern recognition system. The pattern recognition approximation has been analyzed in previous works by Hudgins [41], Zardoshti-Kermani [8] or Englehart [9], among others. The discrete analysis allows multiple functions or degrees of freedom with a reduced set of signals from muscles. This is worthwhile when working with upper-limb impaired patients, as the set of available muscles is reduced.

**Figure 10.**
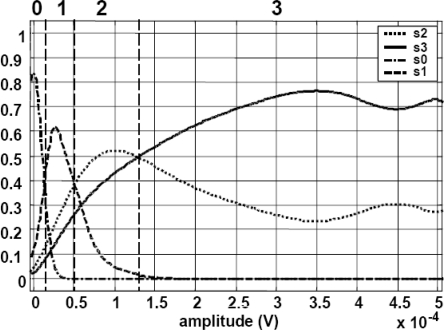
Probability functions P(si|x) and decision thresholds (vertical dotted lines). The thresholds define the regions from 0 to 3, labeled on the top.

The pattern recognition system is designed to distinguish among four contraction levels *s_i_* (*i* = 0…3) in each muscle: rest, weak, medium and strong. The feature extraction system is based on the Zardoshti-Kermani [8] amplitude histogram, characterized by a low computational load. However, such algorithm works with uniform interval separation and it was not considered optimal. Hence, a new algorithm was designed and implemented for this work: *the Bayesian level extractor*. In this algorithm, the interval boundaries are defined by means of four calibration effort patterns for each muscle: rest, weak, medium, strong. The acquired calibration patterns are used to compute the conditional probability functions *P(s_i_|x)*, where *x* is the amplitude of the acquired sample. As shown in Figure 10, the intersections define the amplitude intervals, labeled at the top from 0 to 3. These intervals maximize the probability *P(s_i_|x)* for each state. Thus, the interval separation is applied to create a four-level histogram (hence, a four-component vector) that will feature the pattern. And afterwards, this procedure is extended to the number of muscles under observation, *i.e*., for two muscles, the outcome will be an eight-component vector.

**Figure 11.**
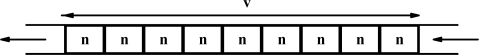
FIFO sliding window.

In real-time operation, the system analyzes the sampled pattern every *n* = 100 ms and samples are buffered. A sliding window technique is applied to the buffer to classify with a first-in first-out (FIFO, see Figure 11) policy. Thus, the analyzed patterns are longer than 100 ms, with an optimum pattern or window size of *v* = 750 ms, as it will be shown later. Hence, every 100 ms. the system processes and refreshes the output in order to maintain the real-time response. A Euclidean distance classifier has been implemented. The basis feature vectors are obtained from the calibration procedure. Thus, for two muscles, seven reference states are defined: both muscles at rest, and weak, medium and strong effort for each muscle.

## 3. Methods

The development of the NTS involved the design of a test collection and the creation of an adaptation protocol. Tests have been carried out in two ways: statically and dynamically.

### 3.1. Static Tests

A static test is a signal recording and, afterwards, a post-processing analysis, *i.e.*, a signal analysis from the recorded signals. This sort of analyses can be found in the works related to MES [9,10]. Hence, the classifier will be analyzed by means of cross-state graphs and classification efficiency tables. Furthermore, the static tests should include the verification of the real-time constraints. Thus, in addition to the standard classification tests, this paper includes a new analysis to evaluate the real-time response: the delay test.

### 3.2. Dynamic or Interactive Tests

As a second stage, the test collection analyzes the UVa-NTS behavior during the real-time operation. These analyses are designated as dynamic or interactive tests. Thus, once the static tests are considered valid, the signal processor is included in the UVa-NTS real-time framework. Then, real-time interactive tests are performed with users. The design and application protocol for the interactive tests is performed in two steps: (i) the UVa-NTS is evaluated with healthy subjects at first; (ii) once a rapid adaptation is established, the UVa-NTS is evaluated with impaired subjects.

The evaluation procedure is performed by means of an adaptation protocol. It starts with the basic signal viewer: then signal quality is checked. It continues with the state navigator, as an intermediate stage, and, it finishes with the *virtual arm* or the *Myo-Pong* tool, as a more complex tool to be commanded. This protocol is applied both to MES and to MMS.

**Figure 12.**
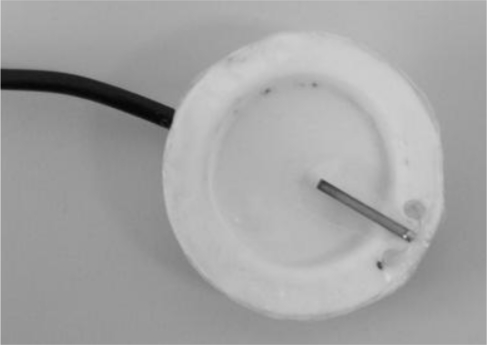
Detail of the MMG electrode.

In order to acquire MES, round surface Ag/AgCl electrodes (1 cm diameter) will be applied, and a bigger Ag/AgCl electrode will be employed as the reference electrode. MMS are acquired by means of two custom vibration sensors built and designed from the BM15015-06HC sensor, from Low Power Radio Solutions, Figure 12. This custom design involved an additional hardware module, included in the UVa-NTS to interface the signals from the vibration sensors. One sensor was placed on the biceps and the other one on the triceps. The UVa-NTS is small and autonomous, as both the computer and the signal-conditioning unit are battery powered. This portability features allow carrying the UVa-NTS to each disabled patient’s home, so tests can be performed in their daily living environment.

## 4. Results

### 4.1. Static Analysis: Classification Efficiency

As previously stated, the envelope detection processor has been widely analyzed in literature. Thus, we will show the static tests performed with the pattern recognition processor. They were carried out with myoelectric signals and with a non-impaired subject. Two muscles were tested simultaneously: biceps and triceps. Seven states or effort levels were defined: rest for both muscles; weak, medium and strong contraction for the biceps; and weak, medium and strong contraction for the triceps. Strong contraction can be near MVC but, in order to get a comfortable operation, a lower and more sustainable non-fatiguing contraction level than MVC was preferred. Contractions were isometric at constant torque. The applied torque for each contraction is shown in Table 1.

**Figure 13.**
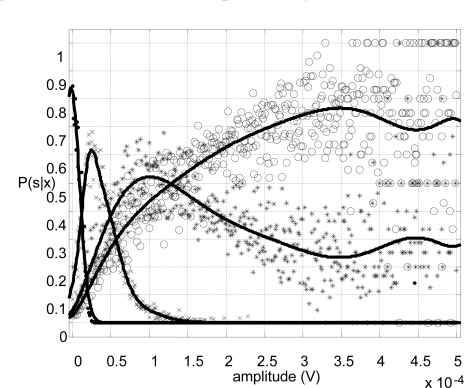
Estimation of the probability functions for each state *s_i_*.

From the calibration patterns, the probability functions were estimated. Figure 13 displays the cloud of samples that allowed the probability estimation, as described in the previous paragraphs. The lines show the Gaussian interpolation fitted to the samples for each state *s_i_*.

After the calibration procedure, for each state *s_i_*, a couple of patterns were obtained: one for the biceps and one for the triceps. Each pattern is non-fatiguing and lasts 20 s.

**Figure 14.**
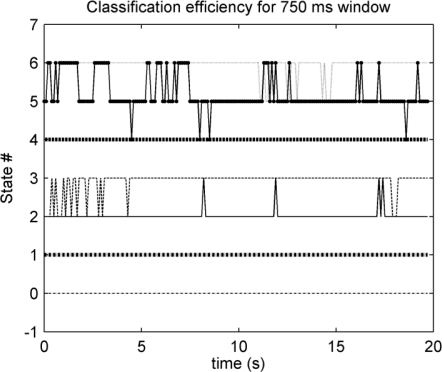
Cross-state graph: classifier efficiency for 7 states (numbered from 0 to 6). State #0 is at rest; #1, #2 and #3 are related to the biceps contraction level, from weak to strong respectively. #4, #5 and #6 are related to the triceps contraction level.

To test the classification efficiency a cross-state graph was plotted. The outcome from this test is shown in Figure 14. The lines, crossing from one state to another, represent misclassification. These results are also displayed in Table 2 for the optimum *v* = 750 ms window: the values out of the diagonal represent the misclassification percentile.

Six cross-state graphs and six classification efficiency tables were generated for different sliding window sizes. The tested sizes were 2 s, 1.5 s, 1 s, 750 ms, 500 ms and 250 ms. As expected, the smaller the size, the bigger the misclassification increment, as the process is not stationary enough.

### 4.2. Static Analysis: Delay Test

The sliding window technique was explained previously, but it is required to choose the proper size *v* for the window in order to achieve real-time performance. Thus, a new test was created to obtain the optimum window size: the *delay test*.

**Figure 15.**
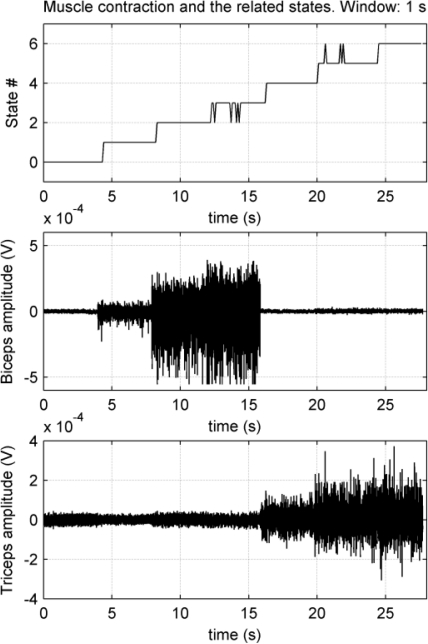
Delay test for a 1 s window size. Top graph displays the classification of the synthetic signal. The two EMG graphs represent the synthetic signals for biceps and triceps.

To implement the test, a synthetic signal was composed with real MES fragments as shown in Figure 15: seven pattern couples (from biceps and triceps) were concatenated to precisely control the state transitions. Each fragment couple represents one state. Therefore the classification of the two composite signals generates a stair graph, as shown on the top graph of Figure 15. Each step or transition boundary was magnified in the computer. Then, the delay between the pattern transition and the classification transition has to be measured.

**Figure 16.**
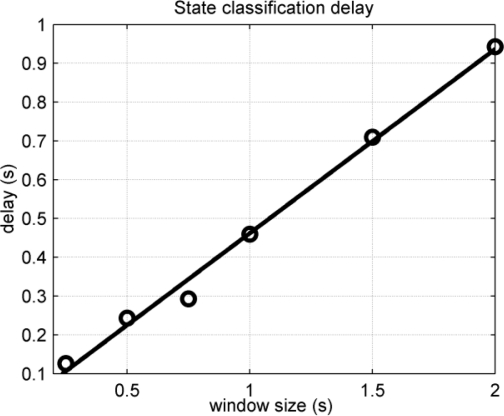
Measured delays linked with an interpolation line.

For a certain window size, a mean delay value was obtained from the six transitions. The process was repeated for each window size, *i.e*., 2 s, 1.5 s, 1 s, 750 ms, 500 ms and 250 ms. The obtained results are displayed in Figure 16.

Three hundred ms will be considered as the delay limit for a real-time response [9]. The measured delay for the 750 ms window was 293 ms and the worst classification percentile was 76% for state #5, as shown in Table 2, but for all the other cases, the classification efficiency is over 94%. Classification efficiency can be increased at the expense of a higher delay, but the algorithm will not be suitable for a real-time response.

### 4.3. Dynamic Analysis: Interactive Test with MES Signals

The implemented graphic tools play an important role in the interactive test. The signal viewer and the virtual arm were developed first. They were tested with the students and the staff from the Telecommunication Engineering School/University of Valladolid, Spain. MES signals were employed for these tests. It was confirmed that the virtual arm was not very intuitive for an untrained healthy subject. It was difficult to transfer the actions from the two muscles (biceps and triceps) to a multi-state virtual arm. Then the state navigator was implemented as an intermediate stage in the training. With this tool, the actions are simpler and closely related to the muscle activity. It was established that the subject follows a progressive learning process: on a first approach, the operator notices the pointer displacements when he/she contracts. Afterwards, the operator learns to discriminate each muscle, by achieving horizontal and vertical pointer displacements. The subject under test realizes he/she can operate the UVa-NTS in a short learning time, and gets more motivated to go on. The final step is to navigate from one state to another and maintain the position for a while in each state. We observed that this procedure made the subject gain self-control for a multi-state device. So, when the virtual arm tool was started, the user was prepared, as most of the adaptation was previously achieved. It was just required to associate each state to one arm action (open/close the hand or extent/flex the arm). The system was tested with five states for maximum state separability. Pronation/supination requires two additional states and a finer control. Thus, the seven-state mode was considered for advanced training.

**Figure 17.**
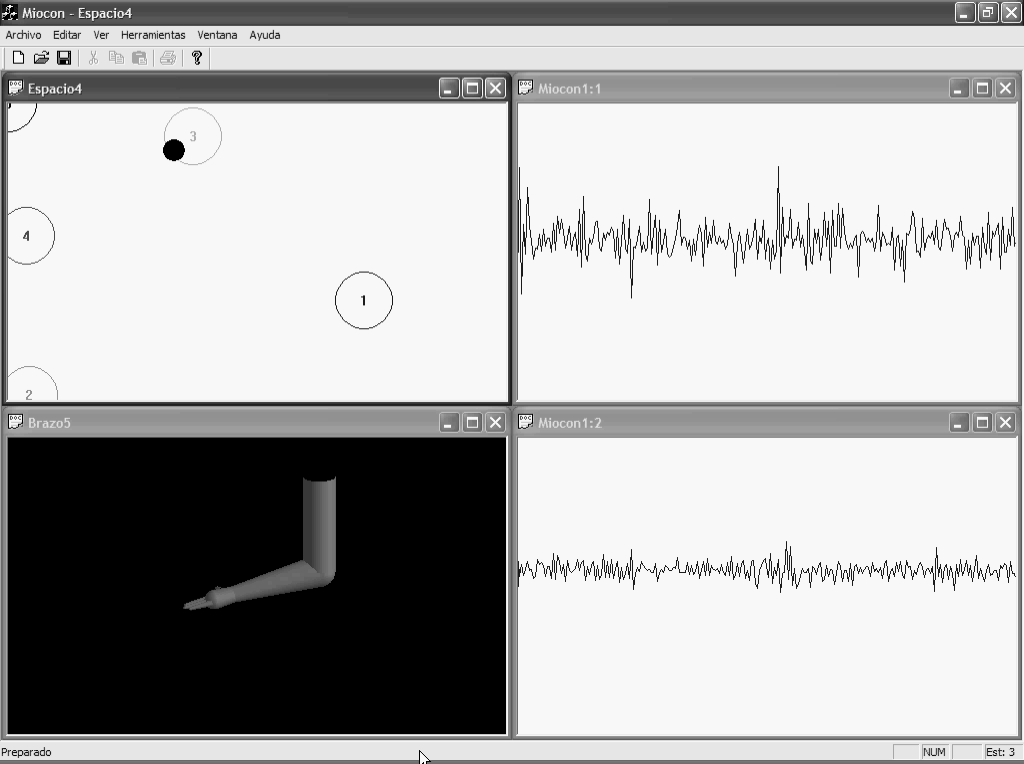
Medium biceps activity and low triceps activity. EMGs from biceps and triceps are shown at the top and bottom graphs respectively. At this moment, pointer in the state navigator is over state #3. State #3 is assigned to hand opening.

**Figure 18.**
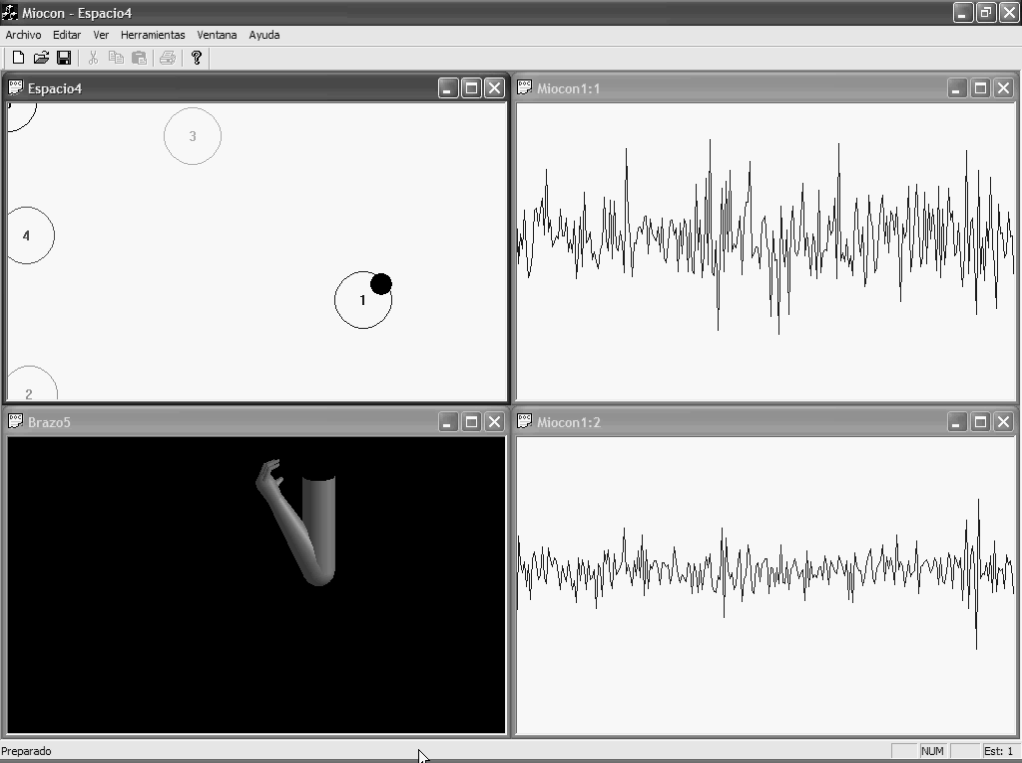
Increased biceps activity (top EMG graph). The detected state is #1, which is assigned to arm flexion.

Figures 17 and 18 display two screenshots during the interactive tests. State #0, on the top left corner of the state navigator represents the rest position. Figure 17 shows the instant when the pointer in the navigator has been positioned over state #3. During the time the pointer is in that state, the hand opens in the virtual arm tool. The associated EMGs from biceps and triceps during the action are displayed on the signal viewer, located on the right side of the figure. In Figure 18 the user has displaced the pointer to state #1 in the state navigator. The biceps activity is increased significantly and thus the pointer is located far from the rest state #0. As shown in Figure 18, an increment can also be noticed in the triceps EMG: this is why the pointer also moves downwards, as the vertical axis is associated with triceps activity.

### 4.4. Dynamic Analysis: Interactive Test with MMS Signals

**Figure 19.**
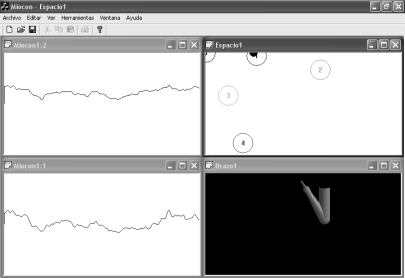
Medium biceps effort, triceps at rest. The signal viewer displays triceps MMG at the top and biceps MMG at the bottom. In this experiment, the viewer time base was originally adapted to EMG. State #1 is assigned to arm flexion.

**Figure 20.**
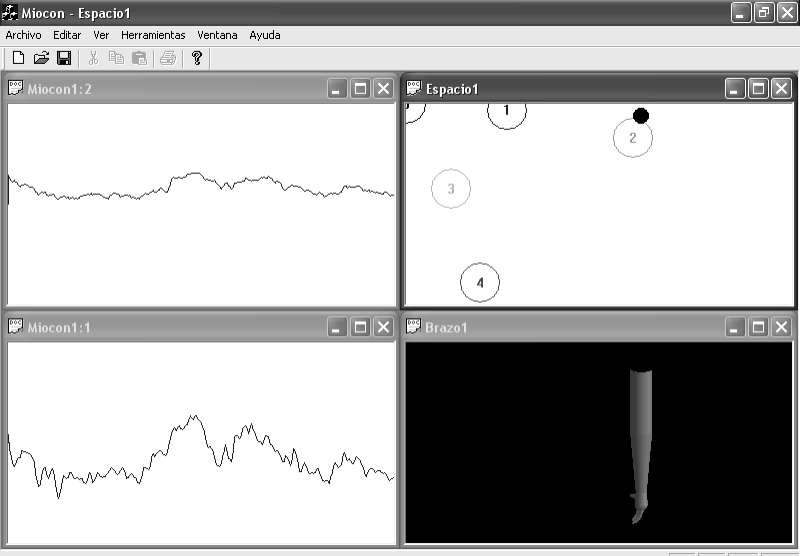
Increased biceps effort. Biceps MMG is at the bottom. State #2 is assigned to arm extension.

We consider this test as a novelty, since interactive real-time tests with MMS have not been found in the literature. This test was performed on healthy subjects at the Telecommunications School. Figures 19 and 20 display two screenshots during tests with MMS. The subject under test exerts two biceps contraction levels and moves the pointer from state #1 to #2. The virtual arm then flexes (state #1) or extends (state #2).

The adaptation protocol applied to MES was also used for the MMS tests. Signal quality and dynamic range were checked with the signal viewer on each subject. Afterwards, the training focused on the state navigator. It was observed that the adaptation to the tool was more difficult: stronger contraction levels were required to achieve a comparable response to MES tests. And a longer training time was required. However, the subjects under test achieved a satisfactory voluntary control with the state navigator after some minutes. Then, they were capable of managing the virtual arm as in the previous tests with MES.

### 4.5. Interactive Tests: Impaired Patients

The system was tested with three selected patients: (i) upper limb disabled, (ii) tetraplegic, and (iii) severe disabled from a cerebral infarction.

**Figure 21.**
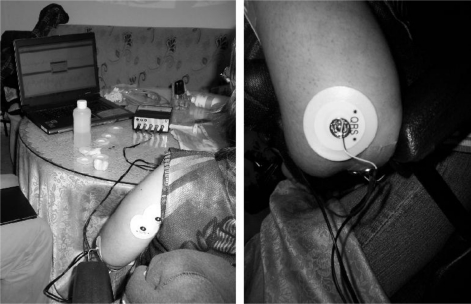
Tests at home with a patient with congenital muscle malformation. An ECG electrode is used as reference electrode. Smaller round Ag/AgCl electrodes (1 cm diameter) are used on the elbow as myoelectric transducers.

Case (i) is shown in Figure 21. She was a 19-year-old girl sat in a wheelchair. She had muscular dystrophy due to a congenital disease that involved progressive muscle deterioration. The patient had congenital upper left limb malformation with the result of no forearm formation. She had an elbow and a residual forearm with certain capabilities, so she was able to hold a needle and inebriate it with the aid of the healthy arm.

A trained healthy subject demonstrated the UVa-NTS operation to the patient, working with MES. Then, the adaptation protocol was applied to the patient: first on the healthy arm and afterwards on the disabled arm. Biceps and triceps were employed. Control of the navigation tool was achieved in less than ten minutes, both with the healthy and the malformed arm. Then the virtual arm tool was easily commanded.

A second test was performed with the residual muscles of the atrophic forearm. The protocol was applied for one signal channel. Then, the patient exerted isometric contractions and, after five minutes, she gained a certain control of the state navigator tool. This encouraged her to go on with the test and, after ten minutes, she was still playing with the tool to improve her skill. During this test, only one direction of the navigator was employed, as only one signal channel was selected for the atrophic muscles.

**Figure 22.**
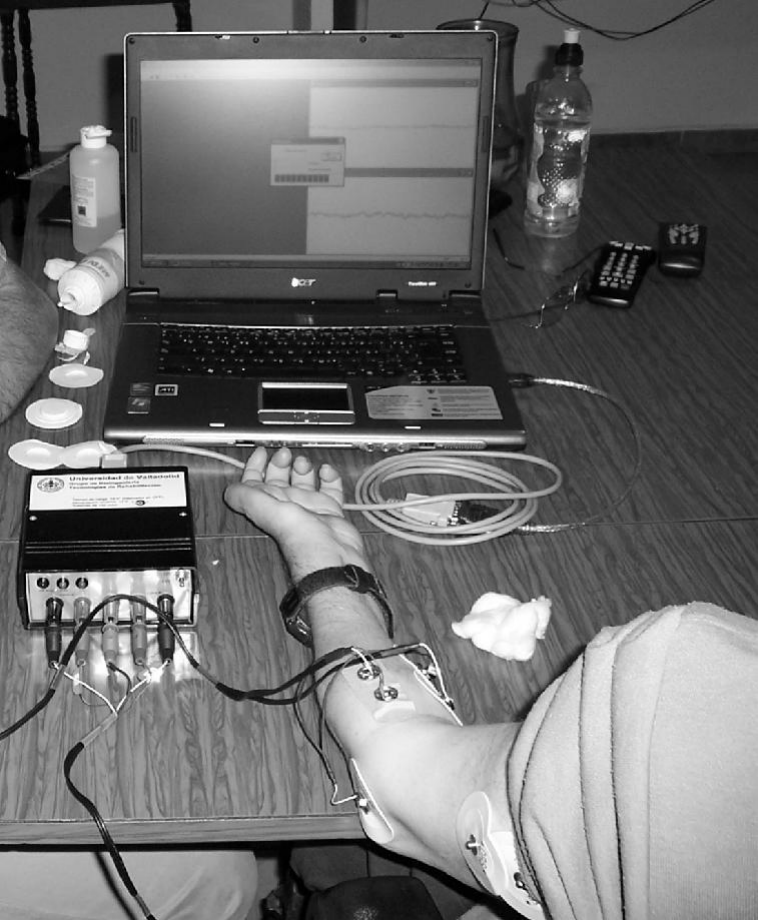
Test at home: patient with tetraplegia during the calibration procedure.

Case (ii) was a 50-year-old C4–C5 partially tetraplegic man, formerly a journalist, and sat in a wheelchair. The impairment was due to a car accident, 25 years before. He had muscular degeneration due to the loss of capabilities in the neuromotor system since the accident. He had a reduced capacity to manage his hand, opening and closing it slightly. So, for this test, flexor muscles of the forearm were selected, as shown in Figure 22. Signals were noticeably weaker than in the previous case, but after five minutes, a certain control of the navigation tool was achieved. The subject insisted on his exercise and after fifteen minutes he realized he was fatigued. In his opinion, it was a very interesting exercise for his atrophic muscles, as he was playing interactively with the UVa-NTS.

Case (iii) was a severely disabled patient, due to a cerebral infarction. He suffers from lock-in syndrome, as his mental faculties are normal and he has recently written a book with and excellent writing style. He cannot speak and can only move his head, his eyes and one finger in his right hand. He can also raise the forearm a few degrees. He spends most of his time with a computer and interacts with it by means of an optic system. Our navigation tool is closely related to the behavior of a mouse pointer. So it was suggested to the patient that the UVa-NTS could act as an alternate man-machine interface to command the computer. The operation of the UVa-NTS was demonstrated to the patient. Afterwards it was tested on his arm, placing electrodes on biceps and triceps. Less than five minutes were necessary to achieve the adaptation to the UVa-NTS. The subject was able to control the state navigator and the virtual arm with a high degree of satisfaction.

In addition, tests are being performed currently at the National Hospital for Paraplegics in Toledo, Spain. In these clinical trials the medical specialists have chosen patients with partial tetraplegia, actually, with C7 spinal cord level injury. The chosen patients have difficulties to move their arms but they have a residual capability to perform movements. In this case, the study focuses in the flexor carpi and the extensor carpi muscle groups. These disabled patients are testing the UVa-NTS platform tools for self evaluation and training, especially the state navigator and the Myo-Pong tools. A custom defined protocol is being applied. These tests are being supervised by a joint team composed of medical specialists and engineers.

## 5. Discussion

The static test allows a precise evaluation of the signal processor. The features of the custom pattern recognition processor have been analyzed: classification efficiency is one of the major quality factors in pattern recognition systems. The characteristic of a real-time platform is the fast response to stimulus, *i.e.*, the real-time response. Both quality requisites have been jointly analyzed to fit the best solution. The classification tests show that the bigger the sliding window, the higher the classification efficiency. However, the delay in the response to the input stimulus is increased with the size of the sliding window, as it was shown in the delay test. Therefore, for a real-time platform, the best solution must fit these two constraints: low misclassification and low delay time. The results show that the 750 ms window size matches the aforementioned constraints for the designed processor.

To make a complete analysis of the system, it is necessary to supplement the static analysis with the dynamic analysis. Subsequently, interactive tests were performed both with healthy and impaired subjects. It was determined that adaptation is decisive to interact with an aid system such as the UVa-NTS. Hence, a protocol with progressive adaptation was designed: it starts with the system demonstration and afterwards the patient continues with the UVa-NTS operation and the set of training tools. It was observed that a successful demonstration creates interest in the patient. Afterwards, the patient operates the UVa-NTS and visualizes his own raw signals (MES/MMS) with the signal viewer. Then, the correlation between the signal behavior and the muscle contraction makes the patient aware of his muscles and the underlying neuromuscular process.

The state navigator was confirmed as an influential intermediary to improve adaptation to more complicated actions. Complexity and effectiveness are well balanced in this tool, so immediate results are obtained in a few minutes. As a consequence, there is a faster adaptation to command more intricate tools, such as the virtual keyboard or the multi-state virtual arm tool.

This progressive adaptation was effective to test new signal sources for voluntary control. Thus, MMS signals have been successfully tested to command the set of tools. Sensitivity with MMS is lower in comparison with MES, as higher contraction levels are required. However, voluntary control has been successfully verified during these tests. Therefore, we believe that sensor technology and signal processing should be more profoundly analyzed, as it was previously discussed with MES [15,16].

The interactive tests have revealed that a friendly environment encourages the user to persist with the training procedures. The patients discover a different way to interact with an augmentative aid, as the residual neuromuscular activity is amplified: they realize that the reduced physical movements from their impaired limbs turn into complete actions in the UVa-NTS. As a result, patient satisfaction is shown when rapid and successful adaptation is achieved.

## 6. Conclusions

Regarding the electronics of the front-end, it is rare to find the whole description of a MES conditioning system in the literature. Detailed descriptions were offered in order to analyze the troubles involved in the complete implementation of a front-end. On the other hand, an affordable design with a reduced number of components has been developed. Such a design does not require any external ADC card, taking advantage of a standard link to the computer and resulting in a portable configuration. Analog, digital and mixed solutions based on switched capacitor filters have been adopted. Hence, most of the signal processing load lays over the computer system. This allows flexibility to embed new signal processing techniques: hence, this adaptability is achieved by an embedded code from a mathematical tool (Matlab). This architecture increases the feasibility to apply new and more complex processing algorithms.

The portability and autonomy of the UVa-NTS have been key features to move it out of the laboratory to perform tests. The platform has been profoundly analyzed and, as it has been stated, two main steps are required to demonstrate the efficacy of a real-time platform: (i) the static analysis and (ii) the dynamic analysis. Both tests assert the efficacy of the commanding system. Then, efficiency is demonstrated in terms of precision and real-time response, *i.e*., classification efficiency and minimum delay time.

The UVa-NTS acts as a training and interface system. Progressive adaptation is a very important fact and it was achieved by creating the adaptation protocol. As a consequence, the designed training tools allow the increase in the exercise complication: from the simple signal viewer and the state navigator, it is possible to progress to the virtual arm, to the virtual keyboard or to the Myo-Pong game.

As a novelty, successful dynamic tests have been performed with MMS. Response is still rough, as considerable contraction level is required. However, the EMI robustness and the inexistence of electric contacts with the body are important features for the design engineers and for EMI polluted environments.

The UVa-NTS acts as an augmentative system, amplifying residual activity. Hence, its versatile conception makes it suitable for extended man-machine interface functions, such as domotic applications, wheelchair command or robotic arm control. Moreover, interfacing with wireless technologies can expand the UVa-NTS applications.

It has been demonstrated that the UVa-NTS constitutes a real-time and multipurpose system, scalable and suitable for research, training, and design of neuromuscular control applications. In addition, new tools and applications can be implemented and new techniques can be demonstrated from the basis of this fully functional structure.

## Figures and Tables

**Table 1.** Contraction and applied torque.

**Contraction**	**Rest**	**Weak**	**Medium**	**Strong**
**Torque (N·m)**	0	4.2	6	9.5

**Table 2.** Classification efficiency for a 750 ms window. States are numbered from #0 to #6. The tested (T) input states are enumerated on the top row. Each clas1 CM sified (C) output state is located on the left column.

C\T	#0	#1	#2	#3	#4	#5	#6
#0	100	0	0	0	0	0	0
#1	0	100	0	0	0	0	0
#2	0	0	98	2	0	0	0
#3	0	0	6	94	0	0	0
#4	0	0	0	0	100	0	0
#5	0	0	0	0	2	76	22
#6	0	0	0	0	0	6	94
